# Supplementary material for: Ataluren binds to multiple protein synthesis apparatus sites and competitively inhibits release factor-dependent termination
Source: Nat Commun. 2022 May 6;13:2413. doi: 10.1038/s41467-022-30080-6 (PMC9076611; doi:10.1038/s41467-022-30080-6)

SUPPLEMENTARY INFORMATION

I. Supplementary Notes 2

II. Supplementary Tables 3

III. Supplementary Figures 6

IV. Uncropped gels 20

SUPPLEMENTARY INFORMATION

Supplementary Notes

Relative value of k_c compared with k_{t1} or k_{p1}

The expected *CC* for the scheme in Fig. 5 at saturating RFC is given by

$$CC_{Sat} = \frac{k_{t1} \cdot k_{p1}}{k_{t1} \cdot k_{p1} + k_c^{\ 2}}$$

The dissociation times, 3 – 5 min (1/0.3 min^{-1} – 1/0.2 min^{-1}), represent $1/k_c + 1/k_{t1}$ as well as $1/k_c + 1/k_{p1}$. Eq. s and the $CC = 0.1$ value from our analysis (Fig. 5a) lead to $k_c \approx 3\ k_{t1}$ and $k_c \approx 3\ k_{p1}$.

Other sites photolabeled by AzAt of potential interest.

We examined AzAt photoincorporation into the 4 small RNAs (CrPV-IRES, 5.8S rRNA, 5S rRNA, and tRNA^{Gln}) contained within POST5, by one-dimensional (1D) urea-PAGE analysis (Supplementary Fig. 3b) giving results presented in Supplementary Table 1. Labeling of CrPV-IRES at 300 μM AzAt showed partial reduction on addition of eRF1.eRF3.GDPNP. In contrast, addition of eRF1.eRF3.GDPNP did not significantly reduce labeling of the other small RNAs. Added ataluren or GJ072 resulted in generally small reductions in the labeling of small RNAs, none of which are clearly significant.

We also carried out RNAse H fragment assay analysis on Fragments IV and V. Fragment IV, CrPV-IRES nts 156-221, contains the U177 and G186 nts, which, as shown by RNA-Seq, have mutation rate-fold changes >2 (Fig. 2d). Fragment V, 26S nts 1413-1476, contains the neighboring nts 26S-A1437 and 26S-A1438 which are located at the apical loop of the H44 of 28S rRNA, near the GTPase association center, and each nt is within 10 Å of ribosome-bound eRF1. Although they each have only 1.3-fold - 1.4-fold mutation rate changes as determined by Laser-Seq analysis, because of their mutual proximity it is not unreasonable to conjecture that they might be labeled from a common site. As summarized in Supplementary Table 1, only relatively minor decreases in AzAt photoincorporation into Fragments IV and V were observed on addition eRF1.eRF3.GDPNP, and no clearly significant changes were observed on addition of either ataluren or GJ072.

We conclude it is unlikely that AzAt photoincorporation into either the small RNAs or Fragments IV and V occurs from a functionally important ataluren binding site.

Supplementary Tables

**Supplementary Table 1. Photoincorporation yields (mole%) into Small RNAs and Fragments IV and V within the Stop-POST5 complex[a]**							
	**Experiment[b]**	**CrPV-IRES[b]**	**5.8S rRNA[c]**	**5S rRNA[c]**	**$tRNA^{Gln}$ [c]**	**Fragment IV CrPV-IRES nts 156-221[d]**	**Fragment V 26S 1413-1476[d]**
PAL, 300 µM AzAt	-	7.3 ± 0.5	0.89 ± 0.10	1.62 ± 0.11	2.3 ± 0.2	3.2 ± 0.8	0.29 ± 0.03
	eRF1/eRF3/ GDPNP	5.4 ± 0.6	0.80 ± 0.02	1.3 ± 0.2	2.2 ± 0.2	2.9 ± 0.6	0.20 ± 0.01
PAL, 30 µM AzAt	-	0.85 ± 0.14	0.25 ± 0.01	0.19 ± 0.01	0.30 ± 0.04	0.23 ± 0.08	0.020 ± 0.003
	Ataluren	0.76 ± 0.05	0.19 ± 0.01	0.15 ± 0.01	0.22 ± 0.03	0.17 ± 0.03	0.034 ± 0.006
	GJ072	0.59 ± 0.14	0.19 ± 0.07	0.15 ± 0.01	0.25 ± 0.03	0.22 ± 0.05	0.019 ± 0.004

[a] Error ranges are ± average deviations, n=2; [b]Added concentrations: eRF1, 2 µM; eRF3, 4 µM; Ataluren, 500 µM; GJ072, 150 µM; GDPNP, 1 mM; [c]1D-urea-PAGE analysis; [d]RNAse H fragment assay analysis

Supplementary Table 2. Native chemical modification on shrimp cyst rRNA detected by RNA-seq

Shrimp rRNA numbering	Yeast rRNA numbering	Human rRNA numbering	Average mutation rate in untreated samples	Reported modification in yeast rRNA[a]	Reported modification in human rRNA
18S-C953	Insertion between 18S-939 and 18S-940	18S-A998	1.00	None	None
18S-G1734	18S-U1723	18S-G1792	0.99	None	None
5.8S-G120	5.8S-U127	5.8S-G115	0.96	None	None
18S-G231	18S-U229	18S-U250	0.95	None	None
26S-C3156	25S-C2870	28S-C4432	0.95	m5C	None
26S-A764	25S-A645	28S-A1314	0.94	m1A	Am
18S-U1706	18S-U1710	18S-C1766	0.86	None	None
18S-U1203	18S-U1191	18S-U1248	0.80	m1acp3Ψ	m1acp3Ψ
26S-U3239	25S-U2953	28S-U4515	0.58	None	None
26S-G1801	25S-G1576	28S-U2480	0.28	None	None

[a] m5C: 5-methylcytidine; Am: 2′ -*O*-methyladenosine; m1acp3Ψ: 1-methyl-3-(3-amino-3-carboxypropyl)pseudouridine

Supplementary Table 3. rRNA nucleotides susceptible to UV irradiation

Shrimp rRNA numbering	Yeast rRNA numbering	Mutation rate fold change (UV/NULL)	Delta mutation rate (UV-NULL)
26S-415	25S-394	26.4	0.043
26S-420	25S-399	22.8	0.018
26S-836	25S-714	20.7	0.036
26S-1833	25S-1602	16.7	0.027
26S-422	25S-401	14.6	0.019
26S-3008	25S-2723	13.9	0.092
26S-459	25S-429	13.5	0.060
26S-3092	25S-2807	13.1	0.018
26S-2458	25S-2190	12.9	0.043
18S-120	18S-121	12.7	0.060
18S-454	18S-449	12.5	0.032
26S-391	25S-370	11.7	0.033
28S-1688	25S-1495	10.7	0.027
28S-3010	25S-2725	10.1	0.054

Supplementary Table 4. RNAse H oligonucleotide sequences for Fragments I – V		
RNAse H Fragment	Oligo DNA sequences 5' – 3'	Complementary to shrimp rRNA or CrPV-mRNA sequences
I	TCAATTCCTTTAA<br>GAGGTTTCCCGTGTTG	18S 1147 - 1159<br>18S 1204 - 1219
II	CTCTTGCTTAAAACT<br>CTTGGCCGCCAC	26S 3064 - 3078<br>26S 3113 - 3124
III	GGCCCGTTCCCC<br>CTCCACAATACCG	26S 2636 - 2647<br>26S 2696 - 2708
IV	CAGAGAGGGCTTC<br>GTCATTGTCTCAC	CrPV-mRNA 156-221
V	CCGACCTCCATGG<br>GCCTCCCATTTTA	26S 1405 - 1417<br>26S 1467 - 1479

Supplementary Table 5. Number of independent determinations, n, for results presented in Fig. 4g				
	[Ataluren], μM			
[RFC], μM	0	200	500	1,000
0.025	2	2	2	2
0.0375	2	2	4	4
0.050	4	2	4	4
0.0625	6	2	4	4
0.075	6	2	4	5
0.100	6	2	4	4
0.200	4	2	2	4
0.400	4	2	2	4

Supplementary Figures

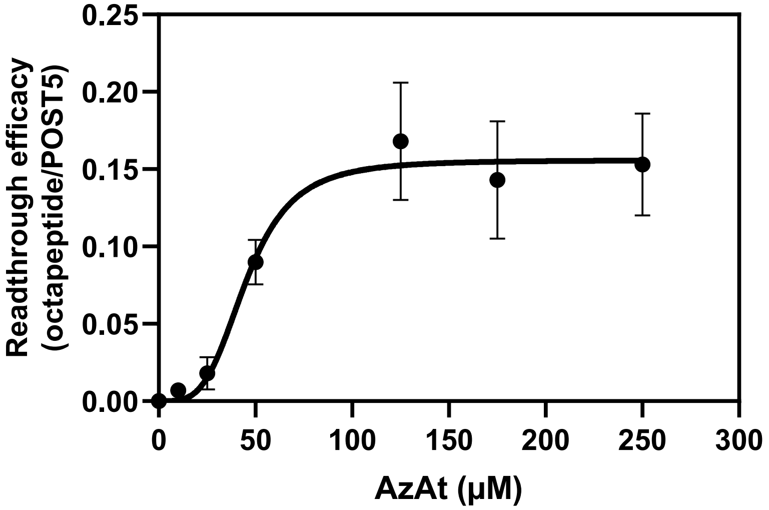

Supplementary Fig. 1. The readthrough efficacy of AzAt measured by PURE-Lite readthrough assay. AzAt is shown to display similar sigmoidal response as ataluren and ataluren-like compounds (Ng et al., 2018), with the apparent EC_{50} of 50 ± 10 μM. The bars represent average deviations for n = 2 independent determinations. Source data are provided as a Source Data file.

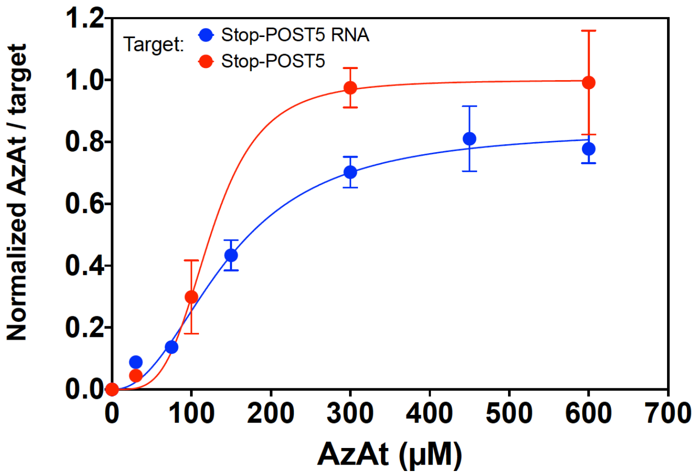

Supplementary Fig. 2. Observed photoincorporation values into Stop-POST5 and the RNA fraction of Stop-POST5 uncorrected for the presence of 80S.IRES in the Stop-POST5 sample as a function of AzAt concentration. Fitting the data to eq (Supplementary 1), where PI is equal to the measured AzAt photoincorporation, yielded a K_A equal to 120 ± 60 μM and a Hill n of 3 ± 1. The bars represent average deviations for n = 2 independent determinations.

$$PI = \frac{PI_{max}[\mathrm{AzAt}]^n}{({K_A}^n + [\mathrm{AzAt}]^n)} \quad \text{(Supplementary 1)}$$

Source data are provided as a Source Data file.

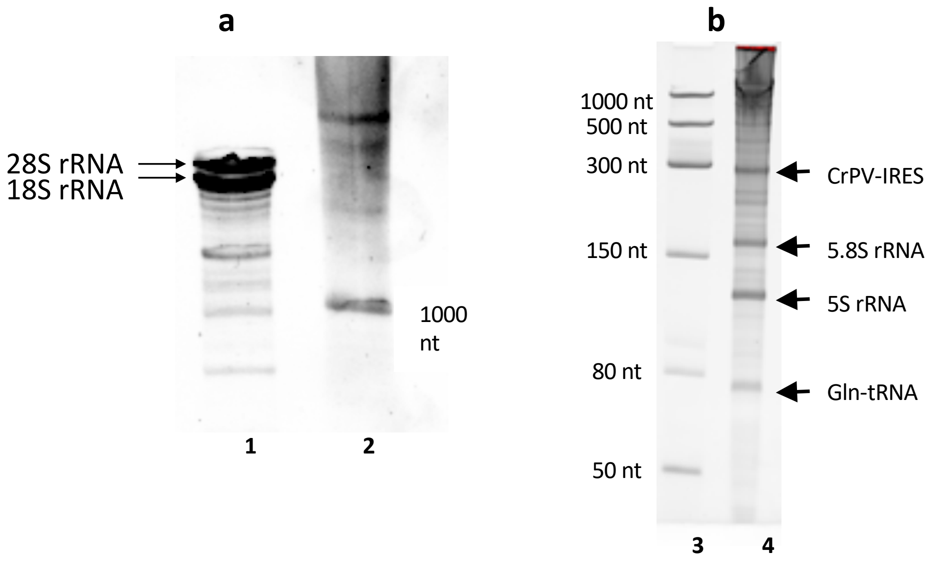

Supplementary Fig. 3. Gel resolution of RNAs. The total RNA of AzAt-photolabeled Stop-POST5 was extracted by phenol-chloroform, and 3 µg of the total RNA was loaded onto each of the gels shown in **a.** and **b.** The bands of 26S and 18S rRNA (**a**) and of each of the labeled small RNAs (**b**) were extracted for measurement. The results for the small RNAs are reported in Supplementary Table 1. **a.** 4% Urea-PAGE denaturing gel. **1.** Extracted Stop-POST5 RNA. **2.** RNA Ladder. **b.** 8% Urea-PAGE denaturing gel. **3.** RNA Ladder **4.** Extracted Stop-POST5 RNA.

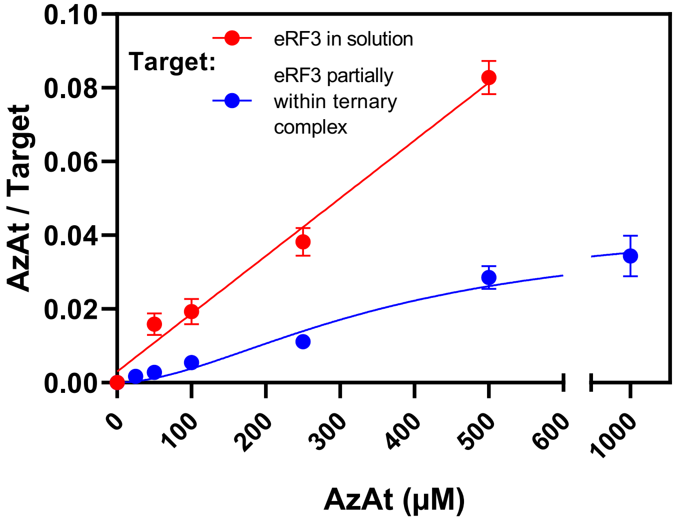

Supplementary Fig. 4 : Photoaffinity labeling of eRF3 in solution (red) or within release factor ternary complex (blue). Both of them show linear response to increasing concentration of AzAt, suggesting photo-incorporation into eRF3 is likely resulted from non-specific binding of AzAt. The bars represent average deviations for n = 2 independent determinations. Source data are provided as a Source Data file.

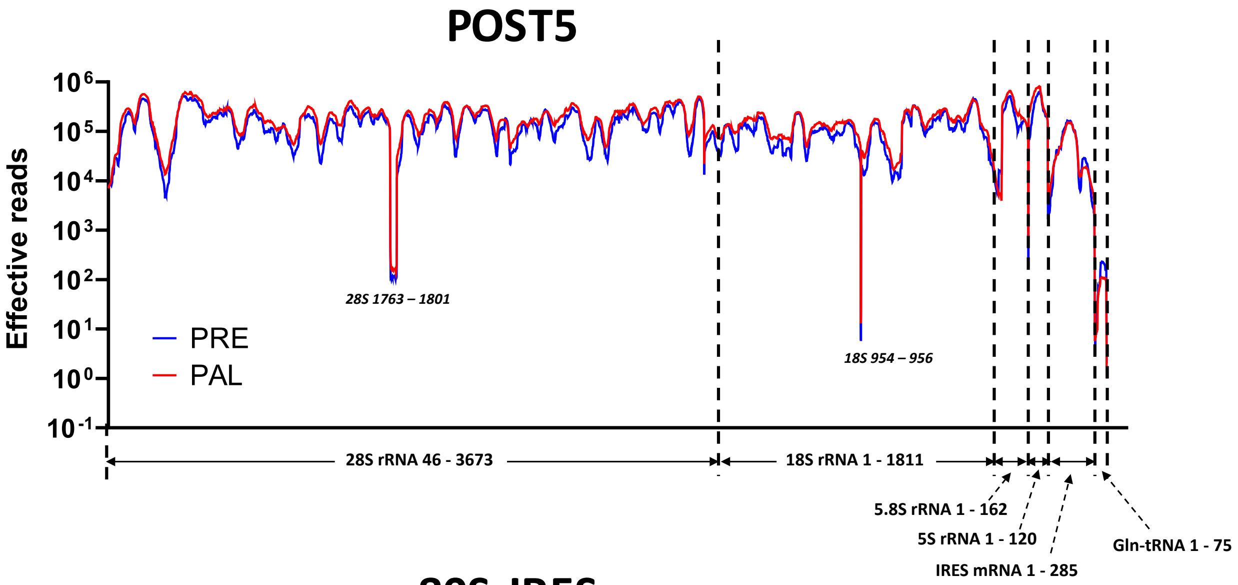

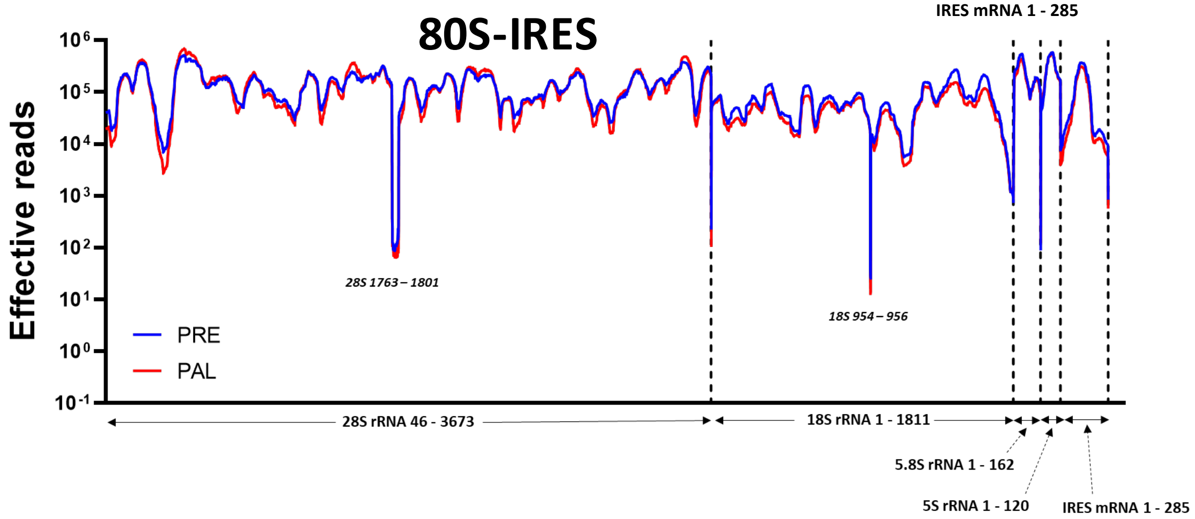

Supplementary Fig. 5. The RNA-seq coverage is shown as the effective reads per nucleotide. PRE: treated with 300 µM prephotolysed AzAt; PAL: treated with 300 µM AzAt. 26S-rRNA nucleotides 1-45 were unable to be mapped due to the lack of sequence information. Regions that are not sufficiently covered by sequencing are annotated (26S 1763-1801, 18S 954-956). Gln-tRNA is not covered by the RNA-seq due to its short length sequence.

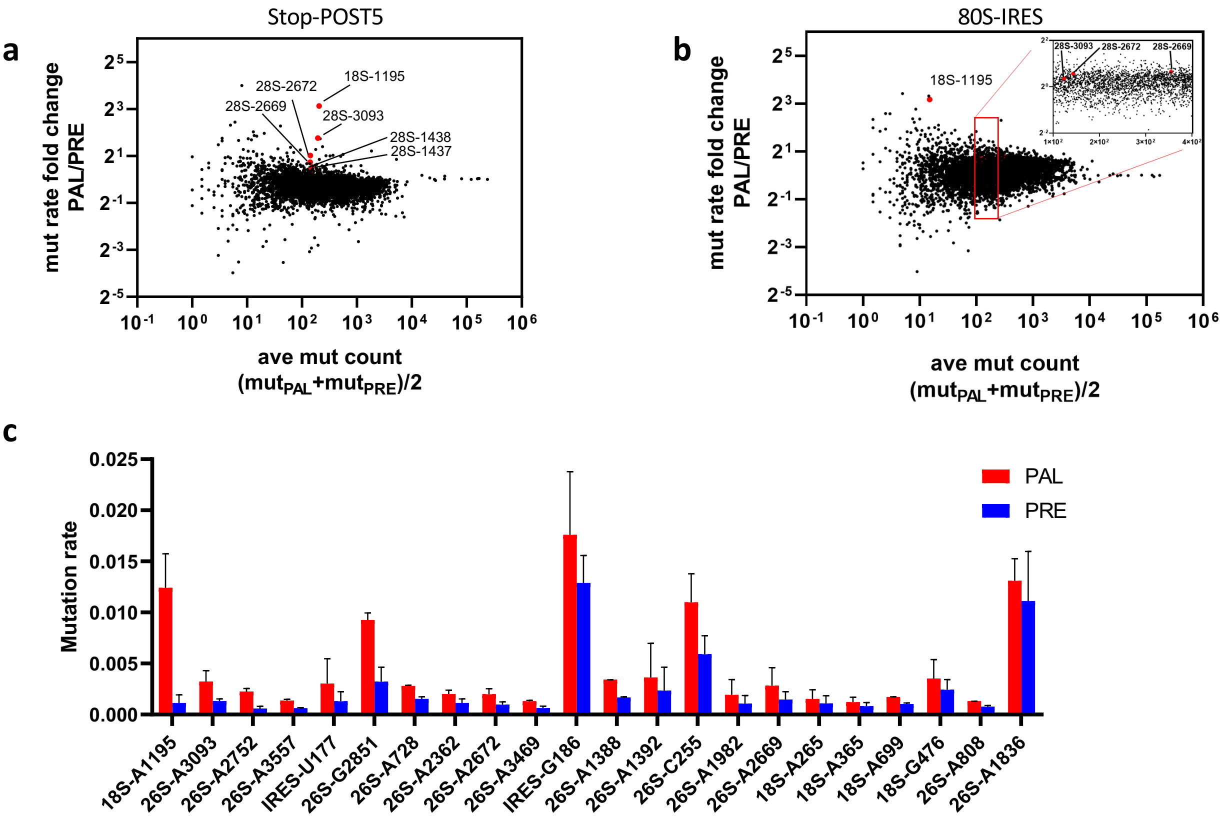

Supplementary Fig. 6. LASER-seq assay to identify putative AzAt photo-incorporation sites. (**a** and **b**): MA plot shows the mutation rate fold change of PAL over PRE as a function of average mutation counts of PAL and PRE for Stop-POST5 and 80S-IRES respectively. Nucleotides of interest are highlighted in red. (**c**) Reproductivity of the mutation rates of the selected 22 nucleotides. Two independent PAL LASER-seq assays were performed with the same condition. The PRE LASER-seq assay was done with or without re-photolysis, and little impact was observed with the re-photolysis condition on these 22 nucleotides. The bars represent average deviations for n = 2 independent determinations. Source data are provided as a Source Data file.

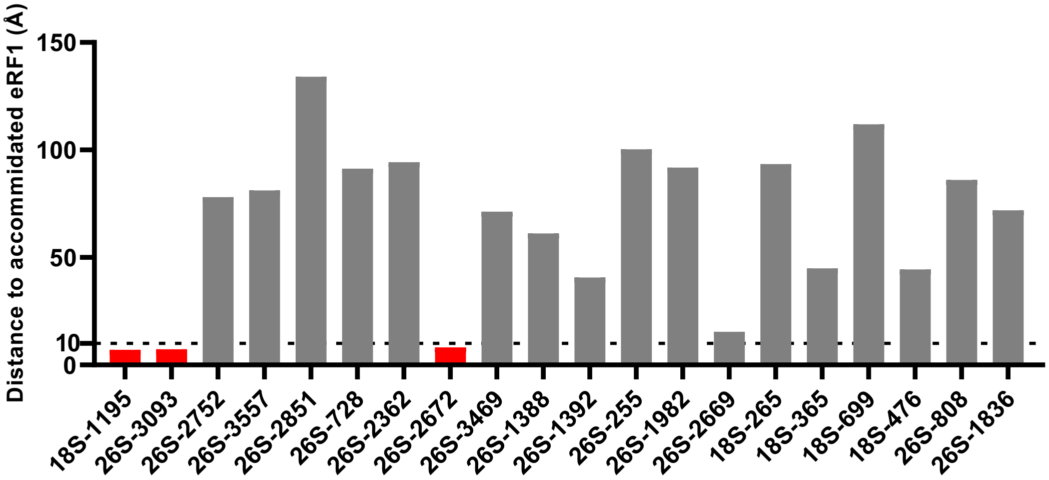

Supplementary Fig. 7. Distance between the selected nucleotides and the fully accommodated eRF1 in cryo-EM structure pdb: 5LZU. Nucleotides that fall within 10 angstrom distance are highlighted in red.

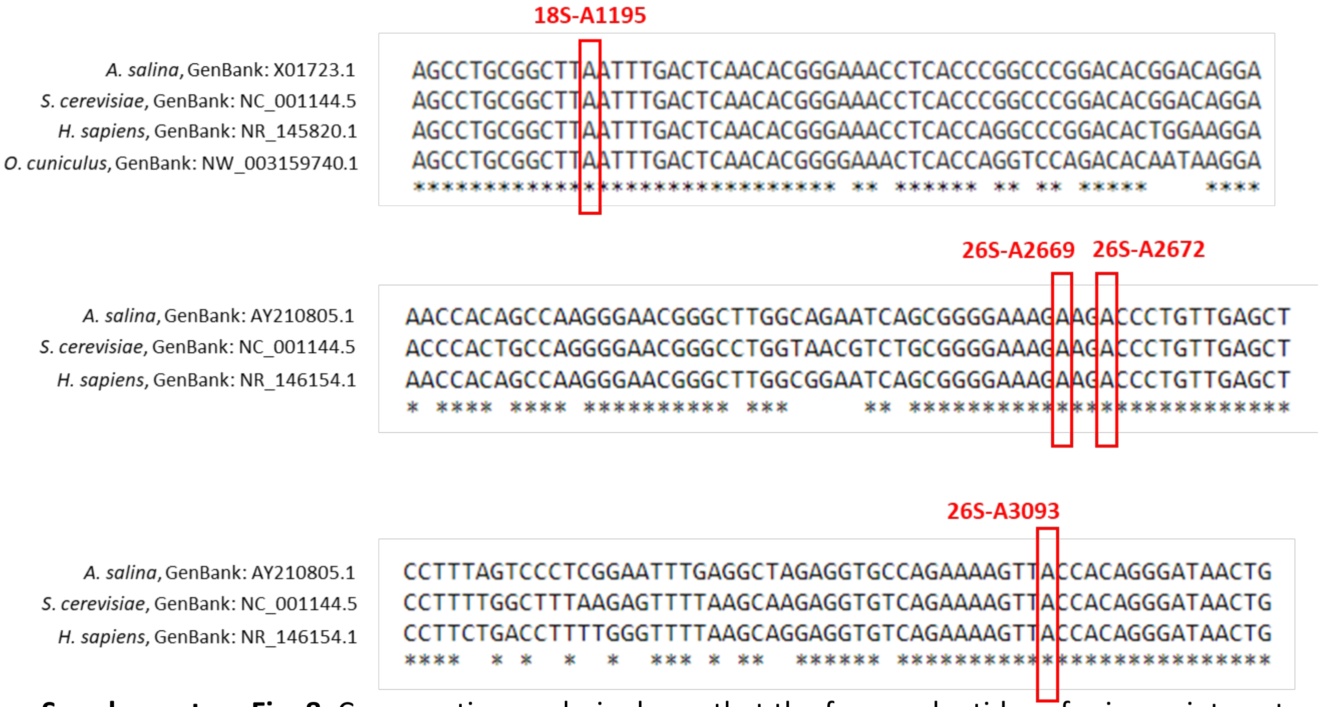

Supplementary Fig. 8. Conservation analysis shows that the four nucleotides of primary interest are all located at highly conserved regions of rRNA. Star symbols indicate identical sequences from different species.

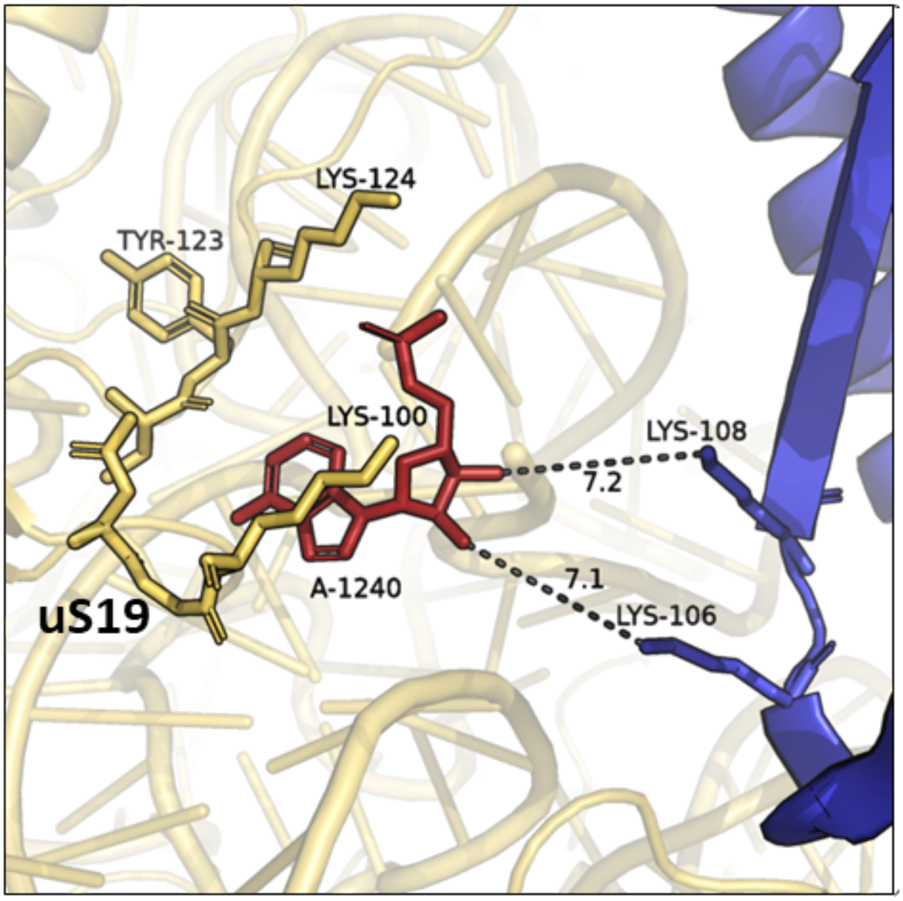

Supplementary Fig. 9. Polar pocket of uS19 formed by Tyr123, Lys124, and Lys100 in rabbit reticulocyte 40S subunit proximal to A1240 (A1195 in shrimp ribosomes) and eRF1 (in blue).

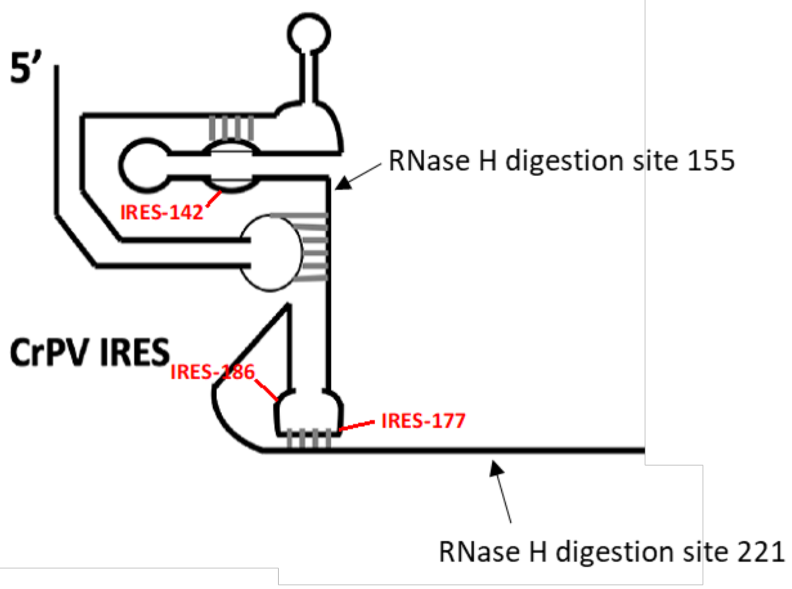

Supplementary Fig. 10. Photolabeled sites and RNase H digestion sites in CrPv-IRES-mRNA

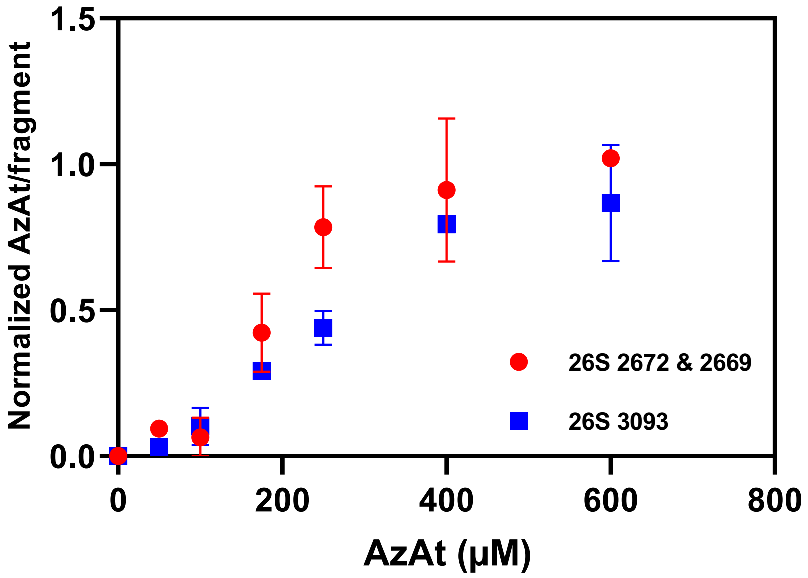

Supplementary Fig. 11. Concentration-dependence of AzAt photo-incorporation into Fragment II (including 26S 3093) and III (including 26S-A2672 and 26S-A2669). The saturated values were 0.8% for both Fragment II and Fragment III. -*S*-shaped concentration dependence is evident for both fragments. The bars represent average deviations for n = 2 independent determinations. Source data are provided as a Source Data file.

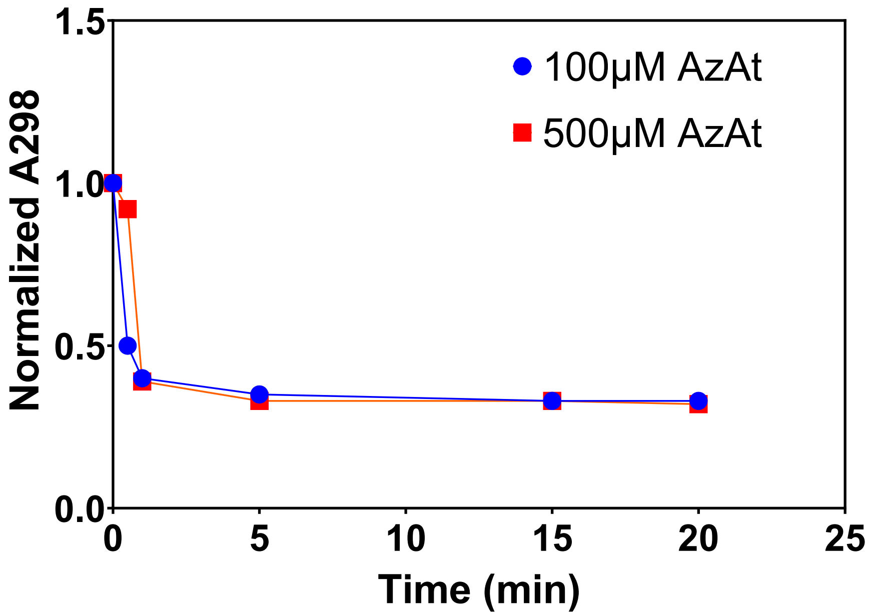

Supplementary Fig. 12. The time course of AzAt photolysis at two different concentrations.

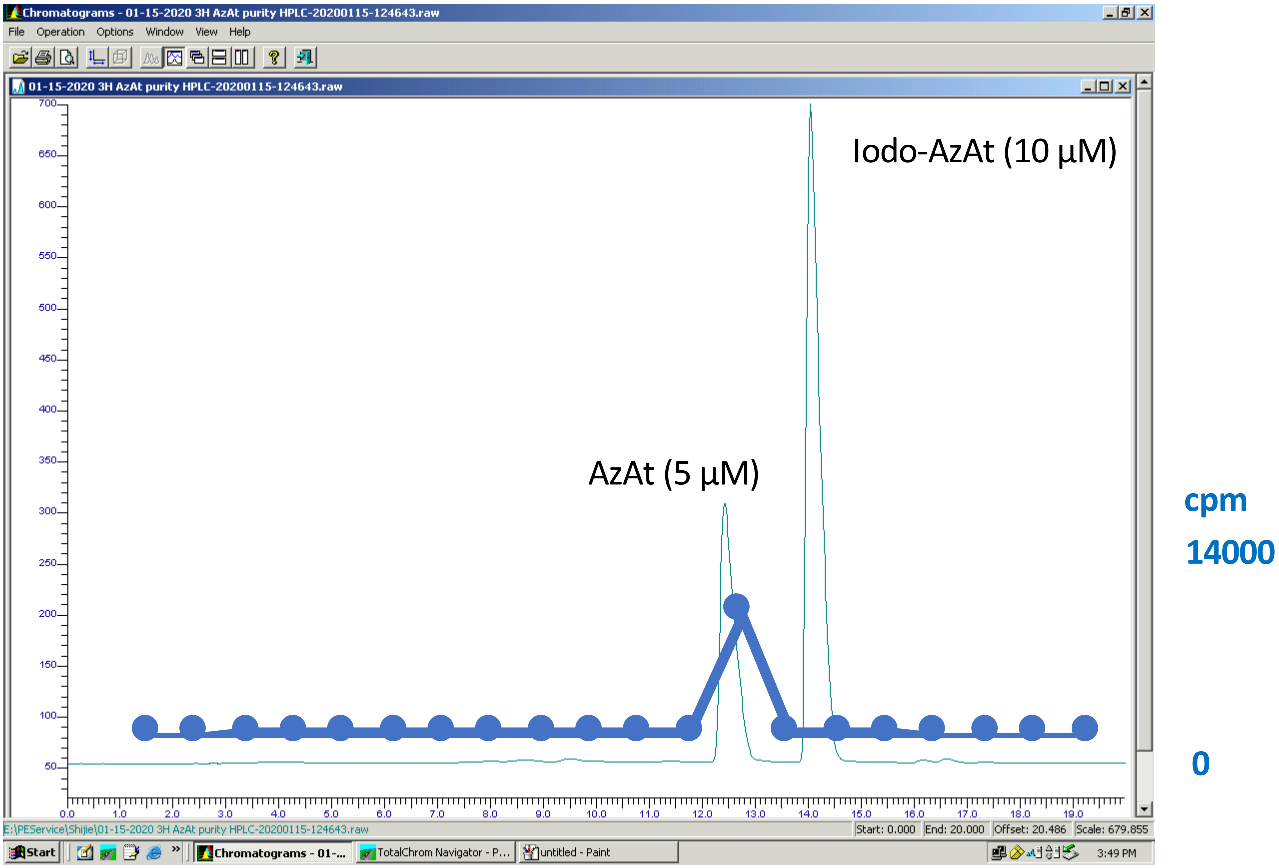

Supplementary Fig. 13. Co-migration of ^{3}H-AzAt and AzAt. The HPLC trace of 5 µM_AzAt and 10_µM Iodo-AzAt which is the precursor of ^{3}H-AzAt, is overlaid onto the radioactivity trace (blue line) of HPLC fractions. Fractions were collected every minute. AzAt and Iodo-AzAt each showed single peaks at the indicated retention times when injected separately.

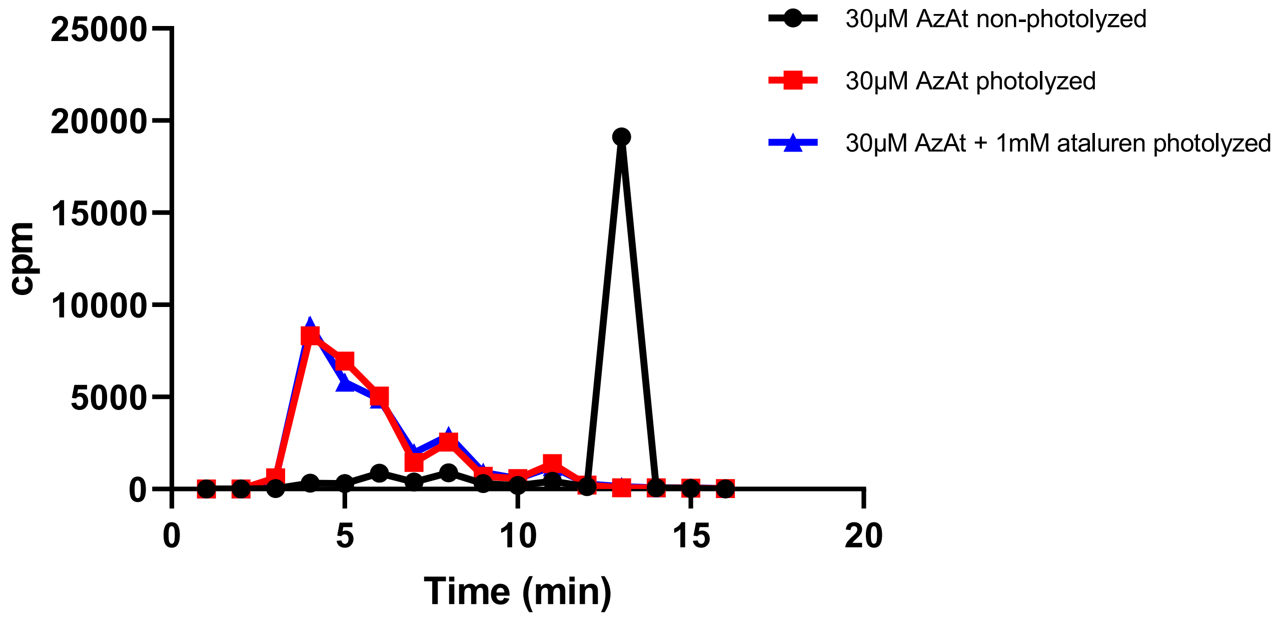

Supplementary Fig. 14. HPLC analysis to check whether there is internal filtering by high concentration of ataluren in the photolysis reaction. Photolysis was performed by 5 min irradiation with 300_nm UV lamp. The result shows that the presence of high concentration of ataluren does not affect the photolysis efficiency.

Uncropped Gels – see Supplementary Fig. 3

a

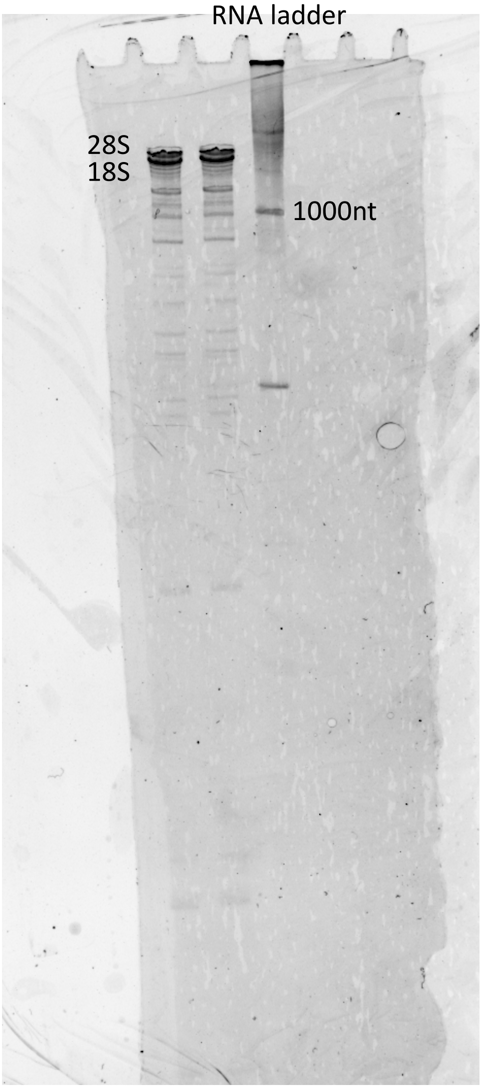

b

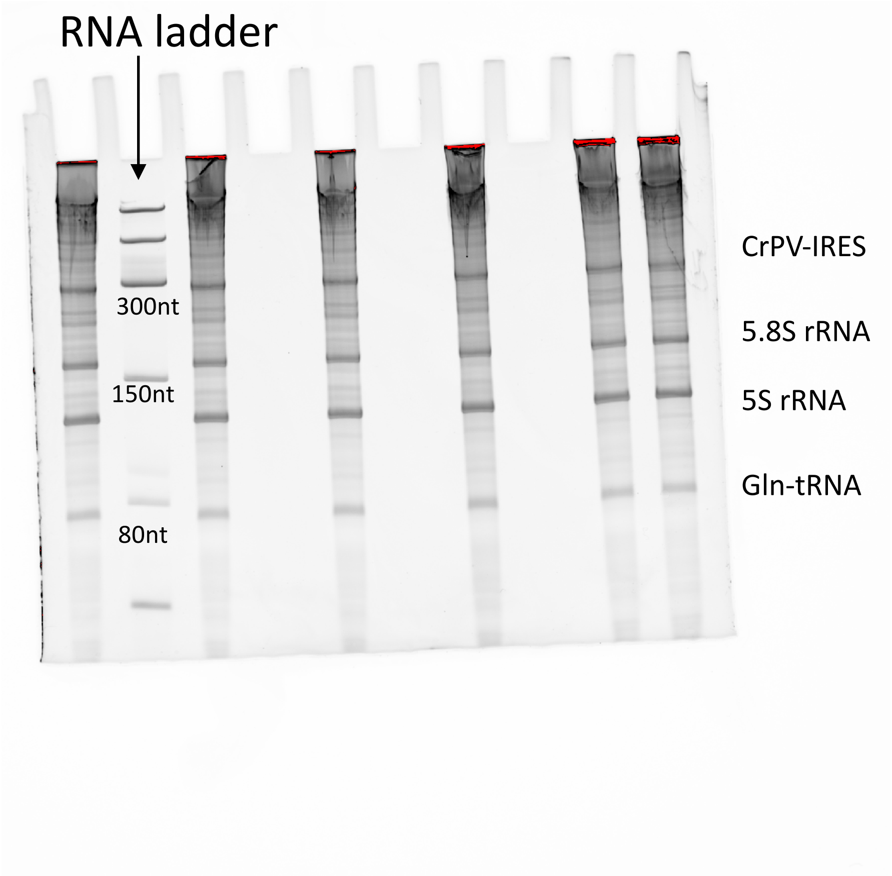

Supplement: Supplementary file 1 — Supplementary Information [file 41467_2022_30080_MOESM1_ESM.pdf]
