# Supplementary material for: Ataluren binds to multiple protein synthesis apparatus sites and competitively inhibits release factor-dependent termination
Source: Nat Commun. 2022 May 6;13:2413. doi: 10.1038/s41467-022-30080-6 (PMC9076611; doi:10.1038/s41467-022-30080-6)
Supplement: Supplementary file 3 — Description of Additional Supplementary Files [file 41467_2022_30080_MOESM3_ESM.pdf]

Description of Additional Supplementary Files

File name: Supplementary Data 1

Description: Raw sequencing reads fastq data files
