# Supplementary material for: Ataluren binds to multiple protein synthesis apparatus sites and competitively inhibits release factor-dependent termination
Source: Nat Commun. 2022 May 6;13:2413. doi: 10.1038/s41467-022-30080-6 (PMC9076611; doi:10.1038/s41467-022-30080-6)
Supplement: Supplementary file 5 — Reporting Summary [file 41467_2022_30080_MOESM5_ESM.pdf]

nature portfolio

Corresponding author(s): Barry S. Cooperman

Last updated by author(s): Mar 11, 2022

Reporting Summary

Nature Portfolio wishes to improve the reproducibility of the work that we publish. This form provides structure for consistency and transparency in reporting. For further information on Nature Portfolio policies, see our Editorial Policies and the Editorial Policy Checklist.

Statistics

For all statistical analyses, confirm that the following items are present in the figure legend, table legend, main text, or Methods section.

n/a	Confirmed	
☒	☐	The exact sample size (*n*) for each experimental group/condition, given as a discrete number and unit of measurement
☒	☐	A statement on whether measurements were taken from distinct samples or whether the same sample was measured repeatedly
☒	☐	The statistical test(s) used AND whether they are one- or two-sided<br>*Only common tests should be described solely by name; describe more complex techniques in the Methods section.*
☒	☐	A description of all covariates tested
☒	☐	A description of any assumptions or corrections, such as tests of normality and adjustment for multiple comparisons
☐	☒	A full description of the statistical parameters including central tendency (e.g. means) or other basic estimates (e.g. regression coefficient) AND variation (e.g. standard deviation) or associated estimates of uncertainty (e.g. confidence intervals)
☐	☒	For null hypothesis testing, the test statistic (e.g. *F*, *t*, *r*) with confidence intervals, effect sizes, degrees of freedom and *P* value noted<br>*Give P values as exact values whenever suitable.*
☒	☐	For Bayesian analysis, information on the choice of priors and Markov chain Monte Carlo settings
☒	☐	For hierarchical and complex designs, identification of the appropriate level for tests and full reporting of outcomes
☒	☐	Estimates of effect sizes (e.g. Cohen's *d*, Pearson's *r*), indicating how they were calculated

Our web collection on statistics for biologists contains articles on many of the points above.

Software and code

Policy information about availability of computer code

Data collection	Next generation sequencing was performed on Nextseq550 and the data was collected using NextSeq System Suite v2.2.0.<br>All the smTIRF experiments were performed on a custom-built objective TIRF microscope (Eclipse Ti-E,Nikon) attached to a EM-CCD camera (Photometrics), and data were collected with a NIS-Element microscope (Nikon) software (vs 5.30.02).
Data analysis	Sequencing reads were processed by bcl2fastq2 v2.20 to generate the fastq files. The universal adapter was trimmed using cutadapt 2.10. Mutational profile was computed using shapemapper2 and star aligner 2.5.2a. Single molecule experiment data were analyzed using custom scripts in Python (3.8) and ImageJ (1.52a) which are available at https://drive.google.com/drive/folders/14Vt_utzLvAqze62S6k37CUQa8xekruMG?usp=sharing<br>The presented figures were prepared in Graphpad Prism 8, MEMLET 1.3, and Python (3.8).

For manuscripts utilizing custom algorithms or software that are central to the research but not yet described in published literature, software must be made available to editors and reviewers. We strongly encourage code deposition in a community repository (e.g. GitHub). See the Nature Portfolio guidelines for submitting code & software for further information.

Data

Policy information about availability of data

All manuscripts must include a data availability statement. This statement should provide the following information, where applicable:

- Accession codes, unique identifiers, or web links for publicly available datasets
- A description of any restrictions on data availability
- For clinical datasets or third party data, please ensure that the statement adheres to our policy

Raw sequencing reads fastq files have been deposited in the BioProject database with the identifier ID PRJNA714648. We stored the source code programs for single molecule analysis in a Google drive with the folllowing link:
https://drive.google.com/drive/folders/1SJnqYL5mWw5O0RrGG_K6L49ztOu03Zjh?usp=sharing

Field-specific reporting

Please select the one below that is the best fit for your research. If you are not sure, read the appropriate sections before making your selection.

☒ Life sciences ☐ Behavioural & social sciences ☐ Ecological, evolutionary & environmental sciences

For a reference copy of the document with all sections, see nature.com/documents/nr-reporting-summary-flat.pdf

Life sciences study design

All studies must disclose on these points even when the disclosure is negative.

Sample size	For all experiments, the number of independent traces are reported in the Figure legends. No statistical analyses were performed to predetermine sample sizes in either the single molecule or ensemble assays.
Data exclusions	No data was excluded.
Replication	All single molecule data were obtained from at least two or three independent experiments. As for single-molecule fluorescence experiments, behavior is stochastic at the single molecule level. Therefore, for kinetic analyses at least 200 traces were used in each single molecule experiments. With these sample size the S.E.M was less than 10% of the mean value. All ensemble experiments were performed between 2 and 11 times, as indicated in the Figure and Table legends.
Randomization	Our studies did not involve participant groups or animals so randomization was unnecessary and not performed.
Blinding	All of our data were used for analysis to avoid bias, so blinding does not apply to our study.

Reporting for specific materials, systems and methods

We require information from authors about some types of materials, experimental systems and methods used in many studies. Here, indicate whether each material, system or method listed is relevant to your study. If you are not sure if a list item applies to your research, read the appropriate section before selecting a response.

Materials & experimental systems

n/a	Involved in the study	
☒	☐	Antibodies
☒	☐	Eukaryotic cell lines
☒	☐	Palaeontology and archaeology
☒	☐	Animals and other organisms
☒	☐	Human research participants
☒	☐	Clinical data
☒	☐	Dual use research of concern

Methods

n/a	Involved in the study	
☒	☐	ChIP-seq
☒	☐	Flow cytometry
☒	☐	MRI-based neuroimaging

March 2021
